# Supplementary material for: Fatty Acid Induced Hypermethylation in the Slc2a4 Gene in Visceral Adipose Tissue Is Associated to Insulin-Resistance and Obesity
Source: Int J Mol Sci. 2023 Mar 29;24(7):6417. doi: 10.3390/ijms24076417 (PMC10094548; doi:10.3390/ijms24076417)
Supplement: Supplementary file 1 [file ijms-24-06417-s001.zip › ijms-2221274-supplementary.pdf]

International Journal of
Molecular Sciences

MDPI

Supplementary Materials:

Supplementary figures

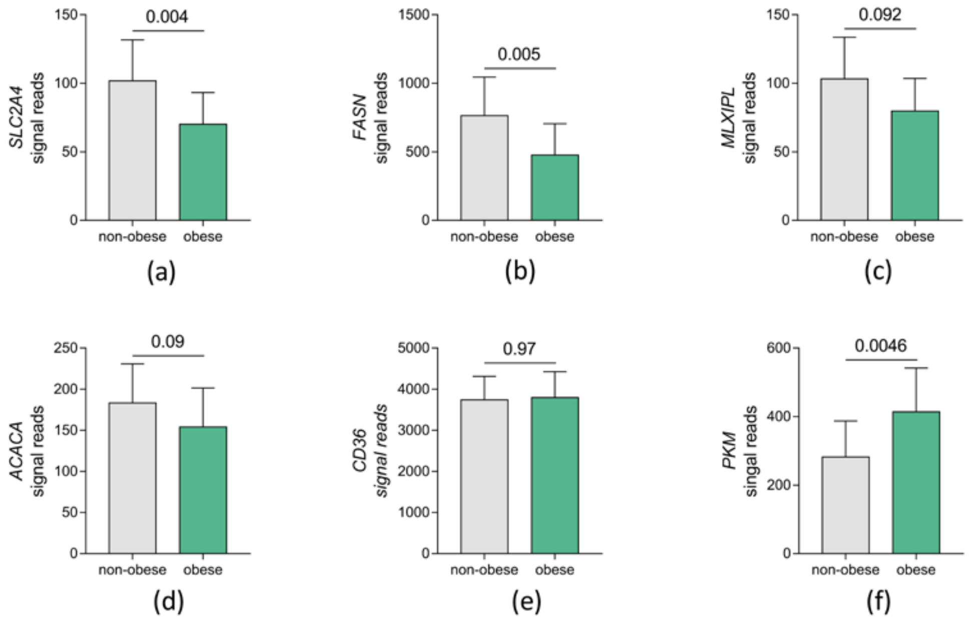

Figure S1. Gene expression from publicly available transcriptomic data (GSE25401) with (a) *SLC2A4*; (b) *FASN*; (c) *MLXIPL*; (d) *ACACA*; (e) *CD36* and (f) *PKM* gene expression, which are highlighted in the volcano plot in figure 1c.

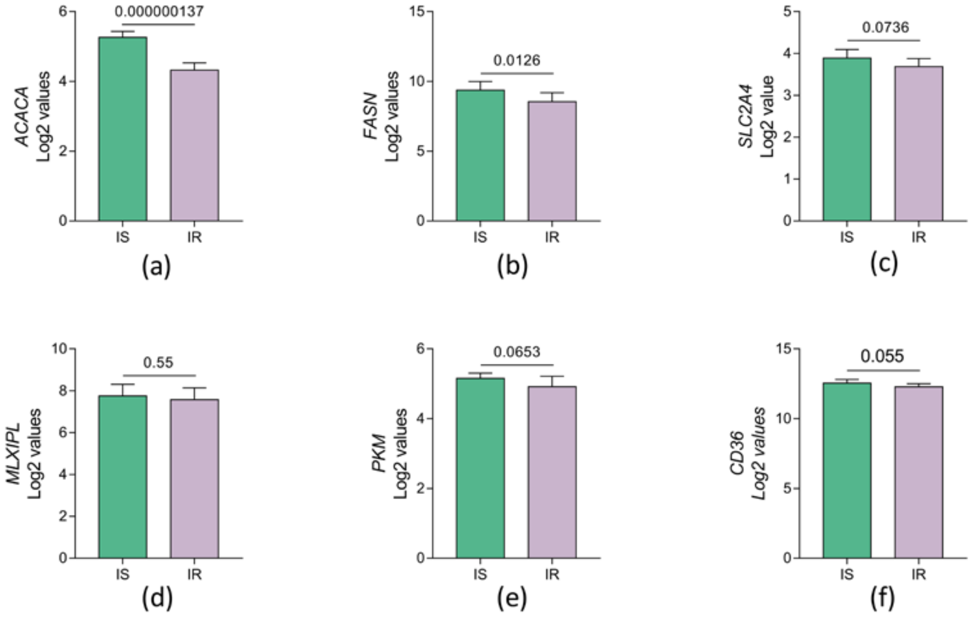

Figure S2. Gene expression from publicly available transcriptomic data (GSE20950) with (a) *ACACA*; (b) *FASN*; (c) *SLC2A4*; (d) *MLXIPL*; (e) *PKM* and (f) *CD36*, which are highlighted in the volcano plot in figure 1d.

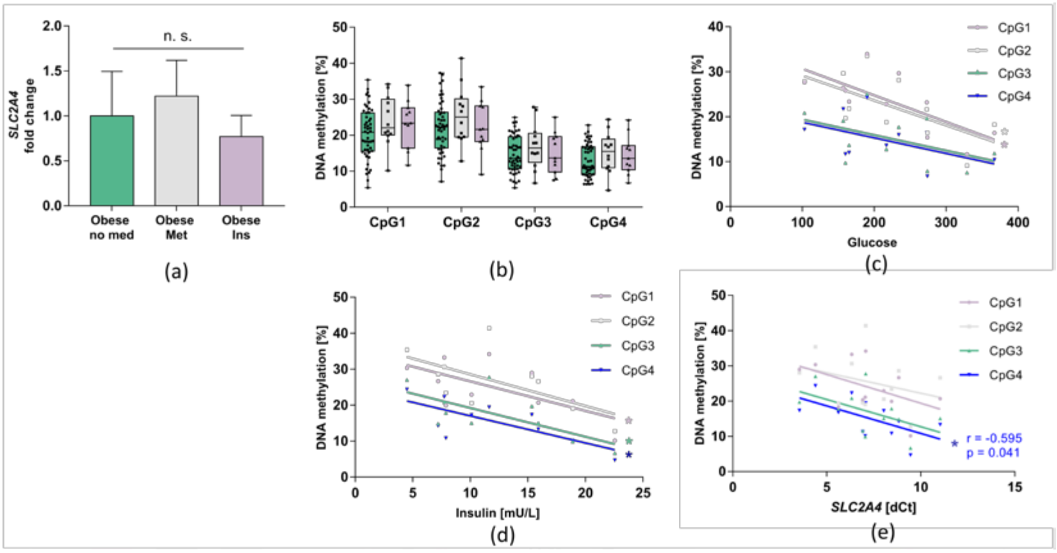

Figure S3. SLC2A4 gene expression and DNA methylation in VAT of human subcohorts. a) SLC2A4 gene expression in VAT of obese subjects with no medication (Obese no med, n = 56), obese with metformin and other oral anti-diabetics (no information on further medication, Obese Met, n = 15) and obese subjects with insulin treatment (Obese Ins, n = 11). b) DNA methylation in VAT of subcohorts. c) Pearson correlation of basal glucose levels with DNA methylation in Obese Ins subjects (n = 11). d) Pearson correlation of insulin levels with DNA methylation in Obese Met subjects (n = 10). e) Pearson correlation of SLC2A4 gene expression with DNA methylation in Obese Met subjects (n = 12). n.s. = non significant.

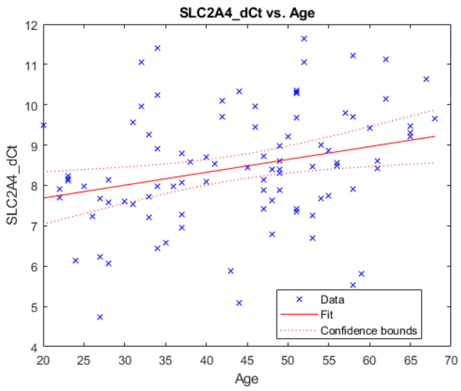

Figure S4. Stepwise regression model excluding SLC2A4 DNA methylation.

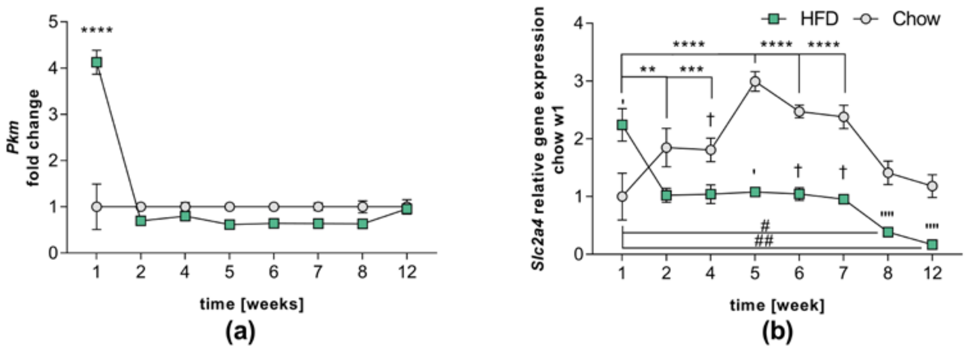

Figure S5. (a) Pkm gene expression in VAT of DIO mice. Mean ± SEM of HFD (n = 7-16) and chow (n = 8-16) is shown. A Two-Way ANOVA using the Sidak correction for multiple testing was performed with **** = $p < 0.0001$. (b) Slc2a4 gene expression normalized to week 1 chow. Mean ± SEM is shown with Chow (n = 8 – 16) and HFD (n = 7 – 16). A Two-Way ANOVA was performed using

Sidak correction for multiple testing (120 in total). */'/# = p < 0.05, **/''/## = p < 0.01, ***/'''/### = p < 0.001, ****/''''/####/ = p < 0.0001. * indicates significances between different chow weeks to chow week 1, # indicates significances between different HFD weeks to chow week 1, ' indicates significances within a week comparing Chow versus HFD and † indicates p < 0.05 before adjusting for multiple testing.

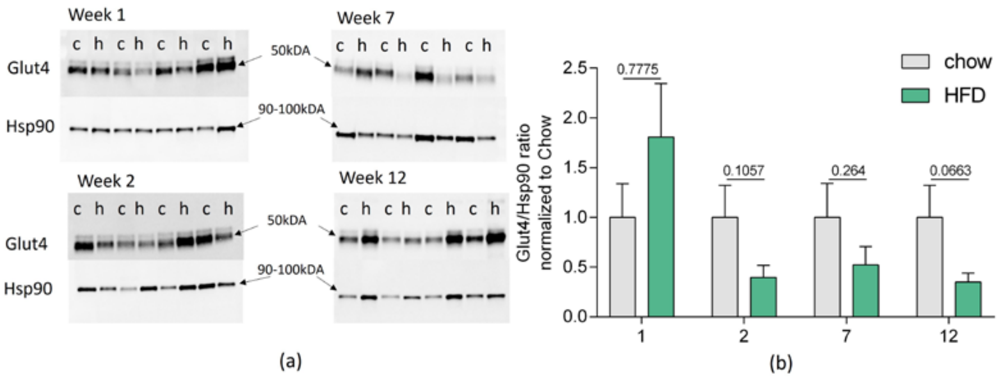

Figure S6. Western blot of VAT from DIO mice a) Representative HRP-signal from four animals per group with h = HFD and c = chow are shown b) Western blot of Glut4 protein in VAT of DIO mice. Mean ± SEM of week 1, 2, 7 and 12 are shown from chow (n = 8) and HFD mice (n = 7 - 8). A multiple t-test was performed.

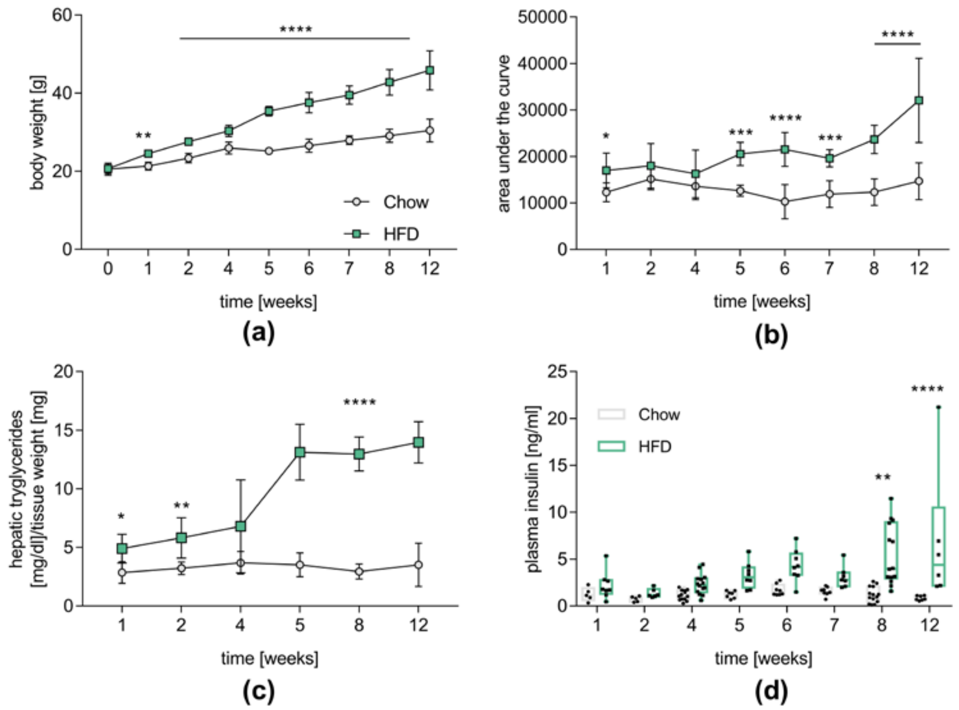

Figure S7. Data of the DIO mouse model (modified [30]). (a) Body weight presented with mean ± SD (chow = 8 – 16, HFD = 7 - 16); (b) Area under the curve of i.p. glucose tolerance tests with mean ± SD (chow = 8 – 16, HFD = 7 - 16); (c) Hepatic triglyceride levels with mean ± SD (chow = 8 – 16, HFD = 7 - 16); (d) Plasma insulin levels with median ± min/max (chow = 5 – 13, HFD = 6 – 15). * = p < 0.05, ** = p < 0.01, *** = p < 0.001, **** = p < 0.0001

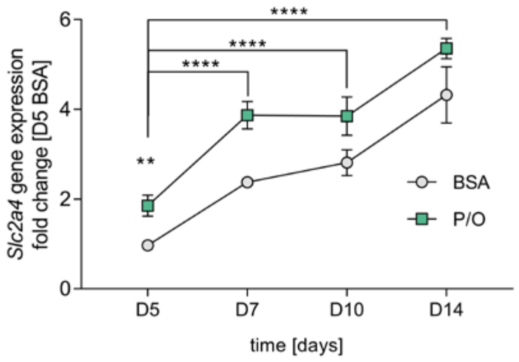

Figure S8. *Slc2a4* gene expression in 3T3 cells normalized to BSA control at D5. ** = $p < 0.01$, **** = $p < 0.0001$

Supplementary tables

Table S1. Estimate values calculated for the stepwise regression model including SLC2A4 DNA methylation seen in figure 1k and formular 2. Number of observations = 94.

Estimates β	Estimates value	P value
β_0	13.281	0.018501
β_1	0.0028359	0.045025
β_2	-0.014897	0.0050012
β_3	0.0086405	0.085903
β_4	0.0117	0.043468
β_5	-0.074511	0.015191
β_6	0.0059353	0.0043084
β_7	-0.013651	0.029534
β_8	0.035458	0.00058659
β_9	-0.032362	0.0032492

Table S2. Primer sequences used for bisPCR and pyrosequencing.

Name	Sequence	Species
f_Slc2a4_ROI_bisDNA	GGAGGGATGGTAGTGAGTGG	mouse
r_Slc2a4_ROI_bisDNA_bio	CCCCCCAACTCAAATTTCCT	mouse
seq_Slc2a4_ROI_bisDNA	GTAGTGAGTGGTGTGA	mouse
f_SLC2A4_ROI_bisDNA	AGGGTAGGAAGGTAGTGTGA	human
r_SLC2A4_ROI_bisDNA_bio	CCACCCCCACTCCTAACC	human
seq_SLC2A4_ROI_bisDNA	GGAAGGTAGTGTGAG	human

Table S3. Primer sequences used for generating insert with overhangs for luciferase assay.

Name	Sequence	Species
f_Slc2a4_ROI_gDNA_over-hangs	CATAAGCTTGAGGAGGGATGG-TAGTGAGTG	mouse
r_Slc2a4_ROI_gDNA_over-hangs	AGTCCATGGGTGGGTG-GAGGGGCAGAG	mouse
